# Platelet-Rich Plasma in Urogynecology: A Case Series

**DOI:** 10.7759/cureus.68004

**Published:** 2024-08-28

**Authors:** Jun Jiet Ng, Sukanda Jaili, John Yen Sing Lee, Nur Ain Yaakob, Vaitheswariy Rao Nalathambi

**Affiliations:** 1 Obstetrics and Gynaecology, Hospital Raja Permaisuri Bainun, Ipoh, MYS; 2 Obstetrics and Gynaecology, Sarawak General Hospital, Kuching, MYS; 3 Obstetrics and Gynaecology, Hospital Bintulu, Bintulu, MYS; 4 Transfusion Medicine and Blood Bank, Hospital Bintulu, Bintulu, MYS; 5 Obstetrics and Gynaecology, Hospital Sibu, Sibu, MYS

**Keywords:** wound healing, vulvodynia, sphincteroplasty, platelet-rich plasma, fecal incontinence, anal sphincter

## Abstract

Platelet-rich plasma (PRP) use in urogynecology is expanding as it shows good results with minimal side effects. We present three new indications of PRP use in urogynecology that have not been previously reported. Our case series demonstrated that PRP improved wound healing and recovery in a woman with chronic obstetric anal sphincter injuries (OASIS) after home delivery, decreased pain in a patient suffering from vulvodynia, and enhanced epithelization in recurrent vaginal stenosis after Fournier gangrene. We have also reviewed patient selection criteria, PRP preparation prerequisites, and techniques. A safe, simple protocol with an optimal platelet yield without using commercial PRP kits is described.

## Introduction

Platelet-rich plasma (PRP) has been widely used in many medical fields such as orthopedics, urology, ophthalmology, aesthetic medicine, sports medicine, and dermatology [1]. However, PRP is a novel treatment in urogynecology. The advantages of PRP therapy are that it is minimally invasive, nonhormonal, uses autologous blood products, and can be performed under local analgesia as day care procedures. To date, side effects reported from PRP treatment, such as pain, bleeding, and infection, are not frequently reported [2]. None of these side effects were observed in our case series. Urogynecology is a subspecialty that treats women with pelvic floor disorders, urinary tract dysfunction, or vulvovaginal problems. Much literature has described the use of PRP for stress urinary incontinence, genitourinary syndrome of menopause, bladder pain syndrome, fistula, vaginal mesh complication, and lichen sclerosis [3]. Our article aims to expand the use of PRP and evaluate its effectiveness in urogynecology. To our best knowledge, there is no literature on the use of PRP in chronic obstetric anal sphincter injuries (OASIS), vulvodynia, and recurrent vaginal stenosis.

## Case presentation

Case 1: chronic obstetric anal sphincter injury

**Figure 1:**
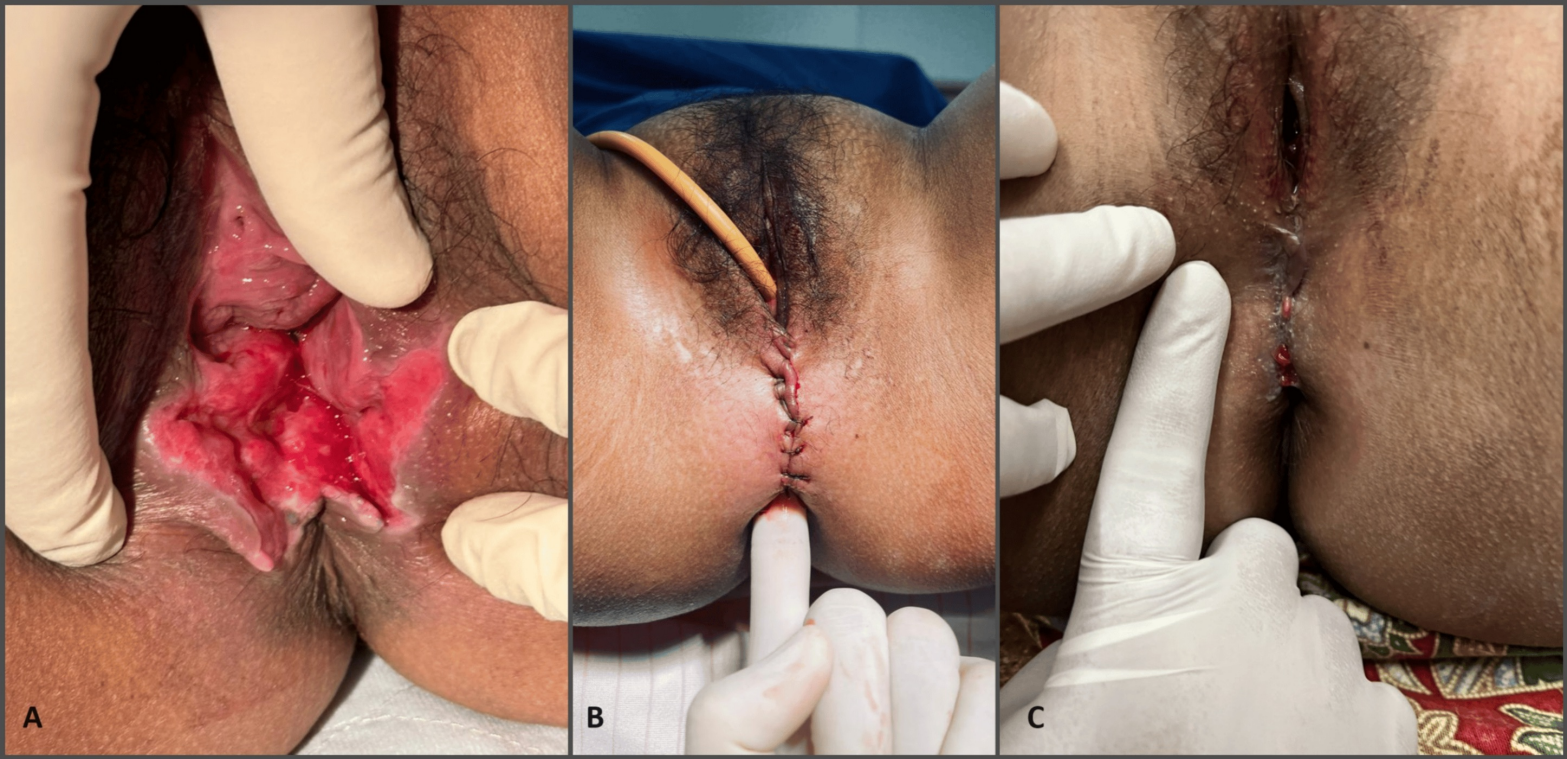
(A) Chronic obstetric anal sphincter injuries after home delivery. (B) Immediate. (C) One month after secondary anal sphincteroplasty, perineal repair, and platelet-rich plasma injection.

A 23-year-old lady, Para 2, presented with complaints of flatus and fecal incontinence one month following home delivery. Her quality of life was being negatively impacted by having to wear sanitary pads. Her Cleveland Clinic Incontinence Score (CCIS) was 13 out of a maximum score of 20. Upon examination, a chronic third-degree tear with a grossly deficient perineum was discovered (Figure 1A). Her anus was patulous, and her anal tone was loose on voluntary squeezing. Despite three months of pelvic floor muscle training, her symptoms did not improve. She underwent secondary anal sphincteroplasty and perineal repair augmented with PRP injection (Figure 1B). The anal mucosa was sutured with polyglactin 3/0 interruptedly; the internal anal sphincter was sutured with polydioxanone 3/0 interruptedly, while the external anal sphincter was sutured using overlapping method with polydioxanone 3/0. Five milliliters of PRP were injected into the anal mucosa and internal anal sphincter and 5 mL into the exterior anal sphincter. At one month's review, her perineal wound healed well with a perineal body of 4 cm (Figure 1C). Her symptoms considerably improved, and her CCIS reduced from 13 preoperatively to two after surgery.

Case 2: vulvodynia and dyspareunia

**Figure 2:**
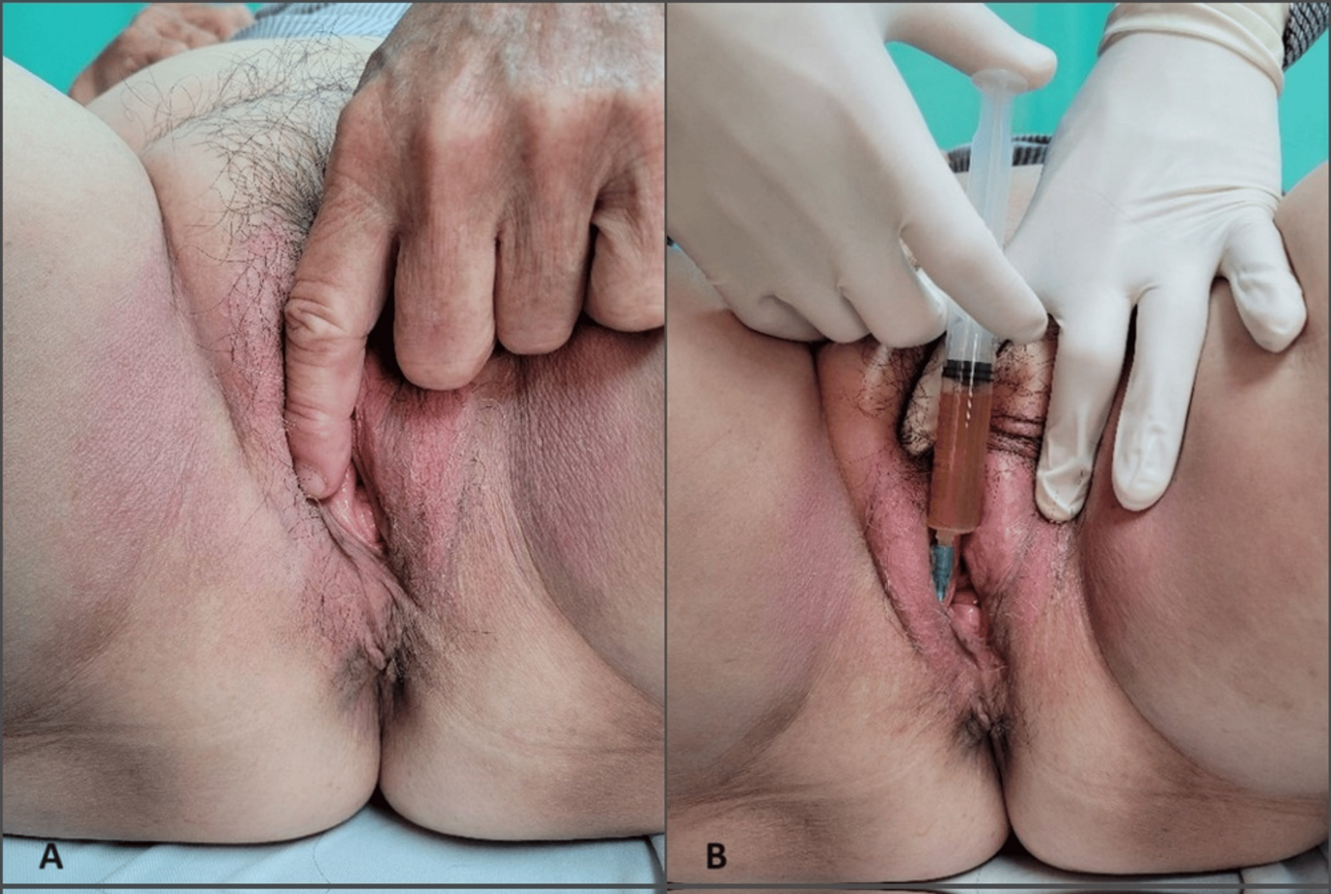
(A) Patient demonstrating her pain site over the vulvar. (B) Platelet-rich plasma injection over the pain site.

A 52-year-old healthy postmenopausal female, Para 2, complained of chronic pain over the vulva for the past 10 years. Her pain score at rest was six on a visual analog scale of 0 to 10 and worsened by local stimulation, including touching, tampons, wearing tight clothing, and sexual intercourse. She denied lower urinary tract and bowel symptoms. She had no underlying comorbidities or past history of sexually transmitted disease or autoimmune disease. External examination revealed normal external female genitalia, and the pain was over the mediolateral episiotomy site at eight o’clock, where she had her vaginal deliveries 12 years ago (Figure 2A). A cotton swab test demonstrated hyperesthesia and allodynia over the pain site. Internal examination found no vaginismus, no levator ani muscle tenderness, and no bladder-related pain. Bulbocavernosus and anal reflex were normal. Despite her attempts with various analgesics, antidepressants, topical therapies, and massages, the pain persisted. Five milliliters of PRP were injected subcutaneously and 5 mL intramuscularly over the area of pain (Figure 2B). At one-month review, she reported improvement in her sexual relationship, and her pain score reduced from six to two.

Case 3: recurrent vagina stenosis

**Figure 3:**
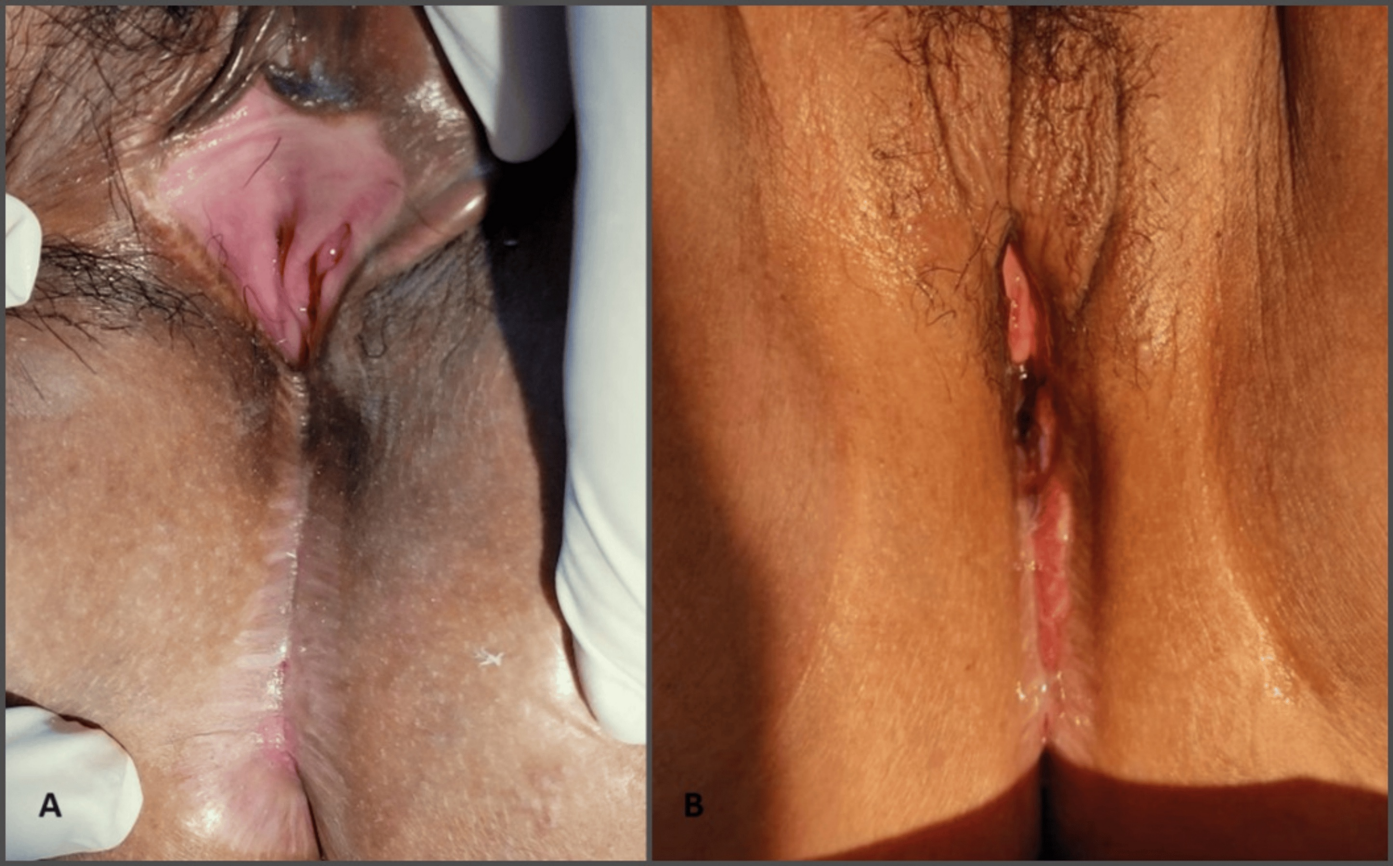
(A) Vagina and anal stenosis after Fournier’s gangrene. (B) After vaginoplasty, Fenton’s procedure and platelet-rich plasma injection.

A 33-year-old diabetic female, Para 5, developed vaginal and anal stenosis after Fournier gangrene of the perineum. It began with genital herpes and progressed into life-threatening Fournier gangrene, requiring extensive surgical debridement and colostomy. The perineum wound healed by secondary intention, and she complained of being unable to consummate with her partner. She was traumatized by pain and bleeding in attempting sexual intercourse. Examination revealed that her vagina and anus were obliterated with well-healed scarred tissue (Figure 3A). Despite having undergone vaginal dilatation and vaginoplasty with amnion two months ago, her vagina canal was partially restenosed after a month, with a narrow vaginal length of 3 cm. Vaginal recanalization and Fenton procedure were carried out, augmented with 10 mL PRP injection into the vagina mucosa and perineal muscle to enhance vaginal epithelization. We decided to augment the repair with PRP in view of this being a repeat surgery. Throughout her treatment, she was taught and emphasized the importance of vaginal self-dilatation after surgeries to maintain the patency of the vagina. At two months review, the vagina was well epithelized with a vaginal length of 7 cm, and she was able to have sexual intercourse with her partner without difficulties (Figure 3B).

## Discussion

Chronic OASIS, vulvodynia, and recurrent vagina stenosis are new indications for PRP use in urogynecology that have not been explored. In the future, we are looking into expanding the use of PRP into other urogynecological conditions such as labial fusion, pelvic organ prolapse, urge urinary incontinence, and sexual dysfunction. The usage of PRP in different age groups, such as adolescents and the elderly, as well as the combination of PRP with stem cells or hyaluronic acid, are prospects that are worth looking into.

The normal platelet value in humans ranges from 150,000 to 450,000 platelets per microliter of blood. PRP is where the concentration of platelets is at least two to five times higher [1]. According to Rughetti et al., to achieve the therapeutic effect of PRP, platelet concentration needs to be at least 1 × 10^6^ platelets per microliter because the stimulation for proliferation of endothelial cells peaks at 1.25 × 10^6^ platelets per microliter, and angiogenesis peaks at 1.5 × 10^6^ platelets per microliter, respectively [4]. The theory underlying PRP treatment is based on the body's natural healing processes, where the first response to tissue injury is to deliver platelets to the injured area. Platelets release a large number of growth factors, such as cytokines, chemokines, lysosomes, and adhesion molecules. All these components can stimulate angiogenesis, chemotaxis, epithelization, cell proliferation, and differentiation, which is an important step in wound healing and regeneration [5]. This explained the pathophysiology behind the PRP use in chronic OASIS (Case 1) and recurrent vaginal stenosis (Case 3). Besides that, it also has anti-inflammatory, analgesic, and neurotonic effects [6]. This explained the pathophysiology of PRP use in vulvodynia (Case 2). The antimicrobial activity of PRP has also been reported [7]. In Cases 1 and 3, PRP was given together with surgical treatment. The success of these surgical procedures has been documented in the literature. Therefore, it is unsure if PRP will have an impact on Cases 1 and 3. This could be our case series' limitation.

PRP can be classified into pure PRP (P-PRP), leukocyte and PRP (L-PRP), pure platelet-rich fibrin (P-PRF), and leukocyte platelet-rich fibrin (L-PRF) [8]. Besides that, there are many variables that can affect PRP compositions and platelet yield: dosages, preparation techniques, application techniques, and additive material. Therefore, it is difficult to evaluate the effectiveness of PRP treatment and to enable its reproducibility. PRP uses the principle of differential centrifugation to divide its components based on their density [9]. In all three cases, we have standardized our PRP preparation method to obtain 10 mL of L-PRP without using commercial kits. Twenty milliliters of blood were redrawn from the medial cubital vein and put into ethylenediaminetetraacetic acid-containing vacuum tubes. The Anitua et al. [10] single-spin closed system centrifuge methodology was followed. A serologic centrifuge (Universal 320, Hettich Zentrifugen, Mülheim, Germany) was used in which the whole blood was centrifuged at 460 relative centrifugal force (RCF) for eight minutes until the whole blood was divided into two layers: PRP and red blood cell layer. The PRP was aspirated out using a 20-gauge needle. Before the procedure, the patient's baseline platelet needs to be checked, as thrombocytopenia is one of the exclusion criteria for PRP therapy. Medications that will impair platelet function, such as nonsteroid anti-inflammatory drugs, antiplatelet, anticoagulants, and steroids, need to be stopped before PRP therapy. Cigarettes, caffeine, alcohol, and vigorous exercise are also not advised [11].

This article informs both patients and healthcare providers about the latest indications for PRP use in urogynecology, as well as provides practical advice. Future studies are needed to standardize the protocol of PRP treatment, which consists of three major steps: blood collection, centrifugation, and PRP storage and delivery.

## Conclusions

The indications of PRP use in urogynecology are expanding, and we have added three new indications in our case series, either as primary or adjuvant treatment, with promising results. PRP is a novel treatment modality in urogynecology that shows good results with minimal risk and side effects. Therefore, PRP can be an alternative treatment for elderly, frail patients who are not candidates for surgery. Although the pathophysiology is not fully understood, PRP has a variety of action mechanisms. Patient selection, pre-procedure requirements, and criteria need to be met to achieve the most benefit of PRP. Standardized PRP preparation techniques and procedures are necessary to assess PRP efficacy and safety, and PRP treatment could be revolutionary. However, it should not be misused and incur additional costs for the patient. Further well-designed studies are warranted to confirm the validity of the reported outcomes.
